# Genetic evolution and phylogenetic analysis of porcine epidemic diarrhea virus strains circulating in and outside China with reference to a wild type virulent genotype CHYJ130330 reported from Guangdong Province, China

**DOI:** 10.1186/s13099-024-00597-w

**Published:** 2024-04-08

**Authors:** Mudassar Mohiuddin, Shengchao Deng, Lisai Zhu, Guiping Wang, Aiqing Jia

**Affiliations:** 1 Guangdong Haid Institute of Animal Husbandry and Veterinary, Guangzhou, Guangdong People’s Republic of China; 2 Guangdong Provincial Key Laboratory of Pig Raising and Disease Control, Guangzhou, People’s Republic of China; 3 Department of Microbiology, Faculty of Veterinary and Animal Sciences, The Islamia University of Bahawalpur, Bahawalpur, Pakistan

**Keywords:** Genetic evolution, Immune protective, Pathogenicity, PEDV, Pigs

## Abstract

During the last decade, porcine epidemic diarrhea virus has detrimental consequences on swine industry, due to severe outbreaks especially in the suckling piglets. In March 2013, an outbreak was reported on a commercial swine farm in Guangdong Province, Southern China. A wild-type PEDV strain named as CHYJ130330 was identified, complete genome was sequenced and deposited in GenBank (accession no. KJ020932). The molecular epidemiological including evolutionary characteristics and pathogenicity assessment were explored during this study with particular interest and focus to develop this candidate strain for new vaccine. The isolates from China pre- and post-2013 shared 96.5–97.2% and 97–99% nt identity respectively with wild-type CHYJ130330 strain which during experimental studies has demonstrated high virulence and 100% mortality in 10^4^ TCID_50_ group piglets within 5 days. The 22 reference strains selected from other parts of the world shared 98–99% identity with our sequence except Chinese (CV777) and S. Korean (vir.DR13, SM98 and atten.DR13) strains sharing 96.8, 97.6, 96.6 and 97.1% identity respectively. The phylogenetic tree revealed most strains reported after 2013 in GII genogroup while the prototype (CV777), S.korean and earlier Chinese (JS2008, 85-7mutant, Atten.vaccine, SD-M, LZC and CH/S) were GI Group. The amino acid sequence of CHYJ130330 E and M protein is highly conserved while ORF3 and N protein having 9 and 17 amino acid substitutions respectively in comparison to CV777 strain. The comparison of full length genome and the structural proteins revealed variations signifying that PEDV variant strains are still the main source of outbreaks in spite of continuous vaccination and also explain the variable trend of large scale outbreaks during this decade as compared to sporadic tendency of disease found before 2010. It is evident from this study that Chinese strains display significant level of mixing with the strains reported from other countries. The strain CHYJ130330 was also adapted successfully to Vero cell line and has shown high virulence in piglets. The information/findings will be helpful to develop a strategy for control of PEDV and have also shown that CHYJ130330 strain has strong virulence and is a more popular clinical strain in recent years, which has the potential to be developed into PEDV vaccine.

## Introduction

Porcine epidemic diarrhea (PED) is an acute, highly contagious enteric viral disease in swine population. Its causative agent, porcine epidemic diarrhea virus (PEDV), is transmitted by oro-fecal route leading to vomiting, enteritis, watery diarrhea followed by severe dehydration and death of animal [1]. Although the disease affects pigs throughout their life but infections are most serious in suckling piglets, with morbidity and mortality rate often reaching 100%. PED was first reported in England in 1971, which later on spread to Europe and even Asia [2]. It was until 2010, when PED was known as a sporadic enteric disease with intrinsic seasonal epidemics occurring in herds in China [3]. However starting in 2010, China experienced a severe outbreak of PEDV that killed more than 1 million piglets in less than two years. Since then, there is a change in the pathogenic potential and severity of the disease with repeated outbreaks in the same herd all the year around, including midst of summer [4]. Consequently, more variant PEDV strains having variations in the sequences from the classical European strain (CV777) have been reported in many Asian countries, including China [5], South Korea, Japan and Vietnam [6, 7].

PED has now been reported in 29 provinces of China, with several outbreaks reported during last ten years [8]. The prevalence rate of PED in China according to various studies varied from 6 to 91% having infection rate varied from 71.43 to 83.47% at different pig farms. According to a survey, the prevalence of PED in Southern China reported in recent ten studies was 41% (1884 positive out of 4562). Moreover, there has been a transition in disease epidemiology to emerge all the year around, with repeated outbreaks and even in vaccinated herds. This unique change in the epidemiology of disease has been suggesting genetic/antigenic alterations in the virus, which needs to be studied to understand the PEDV strains present in China and interpret their phylogenetic relationship with vaccine as well as other reference strains.

Family Coronaviridae includes enveloped virions with single stranded positive sense RNA, which emerged suddenly in 1978 as an enteric pathogen of dogs. PEDV, an alphacoronavirus has full-length genomic sequence approximately 28 kb with unique nested-set arrangement of the subgenomic mRNAs (sgmRNA), possessing four structural (three membrane (Spike or S, Envelope or E, Membrane or M) and one Nucleocapsid or N) proteins and three non-structural (replicases 1a, 1ab and ORF3) proteins, arranged as 5′UTR-ORF1a/1b-S(spike glycoprotein)ORF3-E(envelope)-M(membrane)-N(nucleocapsid)-3′UTR [8, 9]. The 5′UTR is about 291–296 nt, and the 3′UTR is about 334 nt in length. Among structural proteins, S glycoprotein which mediates interaction with the host cell receptors and also induces neutralizing antibodies has been particularly important. The two non-structural proteins, ORFs (1a and 1b) have been responsible for replication and transcription of genome while the third one, encoded in ORF3, is an ion-channel protein. Earlier investigations on the whole genome revealed variations among PEDV field strains and CV777 (vaccine strain).

In China, PED caused by porcine epidemic diarrhea virus has become a serious threat for pig industry, with current vaccines (failure in most cases) becoming ineffective in the prevention of disease. Although, molecular epidemiological studies have been carried out in different regions of China, which were essential for understanding epidemic features pertaining to disease; nevertheless, a more comprehensive studies are required to better understand the genetic variability among local field strains, vaccine strains and other reference PEDV strains. The genetic relatedness and heterogeneity analysis would be helpful to identify the new PEDV variants that are responsible for field outbreaks. The information will be helpful to develop a strategy for control of PEDV and to develop a candidate strain by selection of variant strains for piloting vaccine against PEDV in China. This study was an attempt to understand the molecular characteristics, pathogenicity assessment of CHYJ130330 with future focus to develop this candidate strain into new vaccine.

## Materials and methods

### Ethics statement

The Animal Ethics Committee of Guangdong Haid Institute of Animal Husbandry & Veterinary approved this study. The pigs used in this study for animal experimentation were treated in strict accordance with the requirements of the Animal Ethics Procedures and Guidelines of the People’s Republic of China. Moreover, in vivo experiments were carried out in accordance with ARRIVE guidelines. All animals were euthanized humanely using sodium pentobarbital anesthesia to reduce suffering.

### Sample collection

In March 2013, a 3 day old piglet with vomiting, watery diarrhea and dehydration died at a commercial swine farm, Guangdong Province, Southern China. Fecal sample was collected, labeled and transported to the lab under asceptic conditions for further processing.

### RNA extraction and cDNA synthesis

Viral supernatants from suspected infected fecal specimens were used for sample preparation. RNA was extracted using Omega Viral RNA kit. Reverse transcription of RNA into complementary DNA (cDNA) was done at 42 °C for 45 min by using 2.0 μl of extracted RNA, dNTPs 10 mM each (1 μl), 20 mM Mg^2+^ (1 μl), RNase inhibitor (40U/μl, 0.1 μl), 5 × PCR Buffer (2 μl), Reverse transcriptase enzyme (100U/μl, 0.5 μl), forward Primer (10 nM/ml, 1 μl) and DEPC water (2.4 μl). The cDNA was immediately used for amplification and/or stored at − 20 °C.

### Polymerase chain reaction

For confirmation of PEDV, the ORF3 gene was amplified using primers; ORF3-F (CCTAGACTTCAACCTTACGA) and ORF3-R (CAGGAAAAAGAGTACGAAAA) under the following conditions: an initial denaturation at 95 °C for 5 min, followed by 30 cycles of 95 °C for 30 s, 58 °C for 60 s, 72 °C for 45 s, and final extension at 72 °C for 10 min. The size of the target fragment was 774 bp. The amplified products were subjected to gel electrophoresis and visualized under UV. The PCR product was excised from gel and purified using an agarose gel purification kit. The sequencing confirmed the PCR product as ORF3 gene of PEDV.

### Virus isolation and propagation

PEDV virus isolation was done using Vero cell lines applying slight modifications in the previously used method [10, 11]. Vero cells were seeded in T-25 flask having density 1 × 10^5^ cells, grown at 37 °C for 72 h. Upon infection, media was discarded, and cells were washed twice with phosphate-buffered saline and infected with 1 ml of inoculum. After adsorption for 01 h under constant shaking, the Dulbecco’s modified Eagle’s medium (Gibco, USA) supplemented with 0.3% Tryptose phosphate broth (Sigma, USA), 2 μg/ml trypsin (Sigma), and 1% penicillin–streptomycin (Hyclone, USA) were added and incubated at 37 °C for 24–48 h. Upon observing extensive cytopathic effects (CPEs), the supernatant and cells were harvested by three freeze–thaw cycles, and centrifuged at 10,000×*g* at − 4 °C for 15 min. The supernatants were taken, aliquoted, and stored at − 80 °C. The supernatants were later on used to infect new Vero cells followed by viral propagation using T-75 flasks.

### Electron microscopy (EM) of PEDV particles

When the CPE in the Vero cells was about 90%, harvesting was done for observing virion particles under electron microscope. Briefly, PEDV infected cell culture was frozen and thawed thrice, followed by centrifugation at 10,000 rpm at 4 °C for one hr. Supernatant was filtered through 0.22 µm for removing cell debris and subsequently mixed with polyethylene glycol 8000 (China) at 10–15% final concentration overnight. Mixture was ultracentrifuged at 10,000 rpm at 4 °C for 1.5 to 2 h to obtain pellet of particles. The pellet density gradient centrifugation of 30–40% sucrose was carried out at 30,000 rpm at 4 °C for 3 h. The particles were resuspended in tris-buffered saline solution (TBS) and negative stained using 3% phosphotungstic acid (PTA, pH7.0) followed by blotting and drying. The examination was done using an electron microscope [11].

### Whole genome sequencing

To determine the complete-genome sequence, the isolate CHYJ130330 was sent to Sangon (Shanghai) Co. Ltd.

### Sequence and phylogenetic analysis

The complete genome sequence of CHYJ130330 strain has been available in the NCBI database (Accession No. KJ020932). There were more than 115 whole genome sequences of PEDV in NCBI database. In addition to 22 reference PEDV sequences reported worldwide, 20 sequences reported from China were selected on the basis of region and collection date (Tables 1 and 2). Nucleotide and deduced amino acid (aa) sequences of CHYJ130330 strain and reference strains were retrieved from GenBank and comparatively analyzed. Phylogenetic trees using the entire genomes, and deduced amino acid sequences of S, ORF3, E, M, and N genes were drawn using the neighbor-joining method, MEGA 10 (http://www.megasoftware.net/) with a bootstrap of 1000 replicate datasets.

**Table 1:** Background information of CHYJ130330 and 20 reference strains from China used in this study

S. no.	Strains	Countries	Region/Province	Accession numbers	Year of collection
1	CHYJ130330	China	Guangdong	KJ020932	2013
2	CH/SCMY/2018	China	Sichuan	MH061343	2018
3	H11-SD2017	China	Hubei	MH708243	2017
4	CH/JXJA/2017	China	Jiangxi	MF375374	2017
5	CH/HNZZ47/2016	China	Gansu	KX981440	2016
6	CH/BJ11/2016	China	Beijing	MG546690	2016
7	JS-2/2015	China	Shanghai	KX534206	2015
8	NW17	China	Shaanxi	MF782686	2015
9	CH/HNLH/2015	China	Henan	KT199103	2015
10	PEDV-Hjms	China	Heilongjiang	KY007139	2015
11	CH/GDZH02/1401	China	Guangdong	KR153325	2014
12	85-7 mutant	China	Jiangsu	KX839247	2013
13	PEDV attenuated vaccine^a^	China	Hubei	KC189944	2012
14	SD-M^a^	China	Shandong	JX560761	2012
15	JS2008	China	Jiangsu	KC109141	2008
16	LZC	China	Gansu	EF185992	2006
17	CH/S	China	Guangdong	JN547228	1986
18	CH-SD01/2015	China	Shandong	KU380331	2015
19	CH/ZMDZY/2011	China	Henan	KC196276	2011
20	PT-P96	China	Taiwan	KY929406	2016
21	AH-M	China	Anhui	KJ158152	2011

^a^Cell adapted PEDV strains

**Table 2:** Summary of the background information of 22 reference strains reported worldwide

S. no.	Strains	Country	Accession number	Year of collection
1	CV777	Belgium	AF353511	1978
2	PC273/O	USA	MG837058	2017
3	NB1	South Korea	MF281416	2017
4	PEDV/MEX/QRO/02/2017	Mexico	MH013466	2017
5	PC22A-P140.BI	USA	KX580958	2016
6	KNU-1601	South Korea	KY963963	2016
7	PEDV 1842/2016 ITA	Italy	KY111278	2016
8	SLO/JH-11/2015	Slovenia	KU297956	2015
9	PC22A-P160	USA	KU893873	2015
10	15V010/BEL/2015	Belgium	KR003452	2015
11	Viet/14PED96	Vietnam	KT941120	2014
12	KNU-1406-1	South Korea	KM403155	2014
13	Tottori2/JPN/2014	Japan	LC022792	2014
14	FR/001/2014	France	KR011756	2014
15	COL/Cundinamarca/2014	Colombia	KU569509	2014
16	CBR2	Thailand	KR610994	2014
17	KNU-1305	South Korea	KJ662670	2013
18	Clone=VN/KCHY-310113/2013	Vietnam	KJ960180	2013
19	TC Iowa106 (PV39)-P1	USA	KM392232	2013
20	Virulent DR13	South Korea	JQ023161	2009
21	SM98	South Korea	GU937797	1998
22	Attenuated DR13^a^	South Korea	JQ023162	2003

^a^Cell adapted PEDV strains

### TCID_50_ assay

Vero cells were seeded in 96-well plates at a density of 1 × 10^5^ cells per well and were grown at 37 °C for 24 h. Virus titers were measured by end-point titration in 96-well plates using tenfold serial dilutions of the samples in triplicate for each dilution to determine the amount of virus required to produce CPE in 50% of the infected Vero cells. The 50% tissue culture infectious dose (TCID_50_) per 0.1 ml of virus stock was calculated using the Reed–Muench method [9, 12].

### Experimental infection of CHYJ130330 strain in piglets

Fifteen 3 day old apparently healthy and active piglets purchased from a conventional breeding farm were tested and confirmed negative for PEDV. After acclimatization, piglets were randomly allotted into three groups (5 piglets per group). Each inoculated group was housed in a separate cage. The exclusion criteria included PEDV positive and weak piglets.

Piglets in group 1, 2 and 3 were orally administered with 1 ml/piglet virus solution containing 1.0 × 10^2^, 1.0 × 10^3^ and 1.0 × 10^4^ TCID_50_ of CHYJ130330 PEDV strain. After inoculation, all animals were monitored daily for clinical signs of disease, including diarrhea, vomiting or death.

### Necropsy and histopathology

Postmortem was conducted immediately after the death of piglets. Gross lesions were noted and intestinal samples were collected for histopathology.

## Results

### PEDV CHYJ130330 strain

**Fig. 1:**
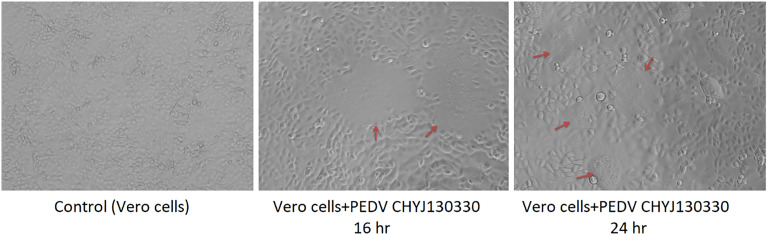
Identification of PEDV CHYJ130330 strain in Vero cells. The panels show cells infected with CHYJ130330 strain (**A**) Control (uninfected) cells (**B**) Cytopathic effects (CPE) at 16 h after inoculation (×10) (**C**) CPE at 24 h after inoculation

The highly virulent CHYJ130330 strain had shown cytopathic effect on Vero cell line after 24 h having characteristic features like fusion of cells and syncytia formation (Fig. 1). The 2–7 day old piglets orally infected with 10TCID_50_ (50% tissue culture infective dose) of this strain had induced severe diarrhea within 48 h [9].

**Fig. 2:**
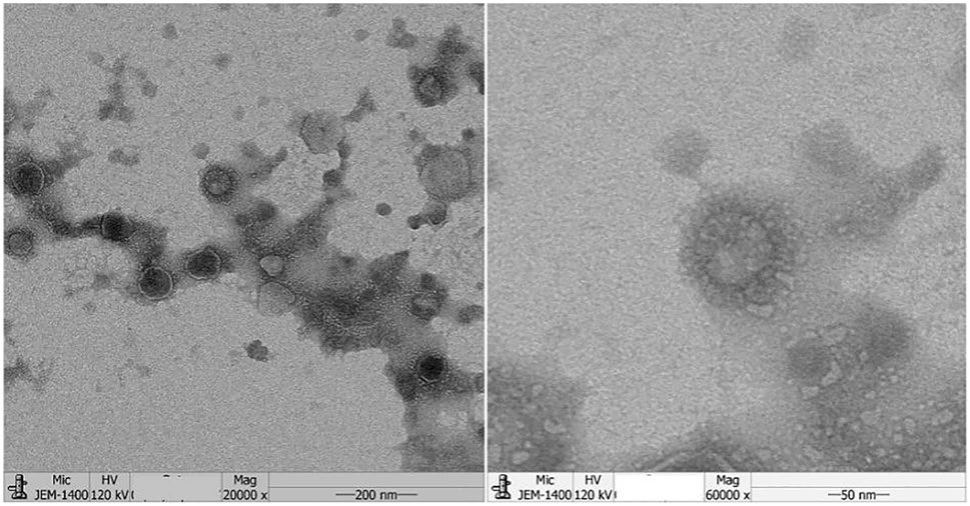
Electron micrograph of purified PEDV virus. Left image is the virus at ×20,000 and right image is magnification at ×60,000 PEDV virions

PEDV (CHYJ130330 strain) particles in Vero cell culture, when observed under electron microscope showed typical Coronavirus particles in cell culture media and in the surface sections of infected vero cells. Circular shaped virions 80–120 nm in diameter with characteristic surface projections were observed (Fig. 2).

### Clinical signs in experimental animals

**Fig. 3:**
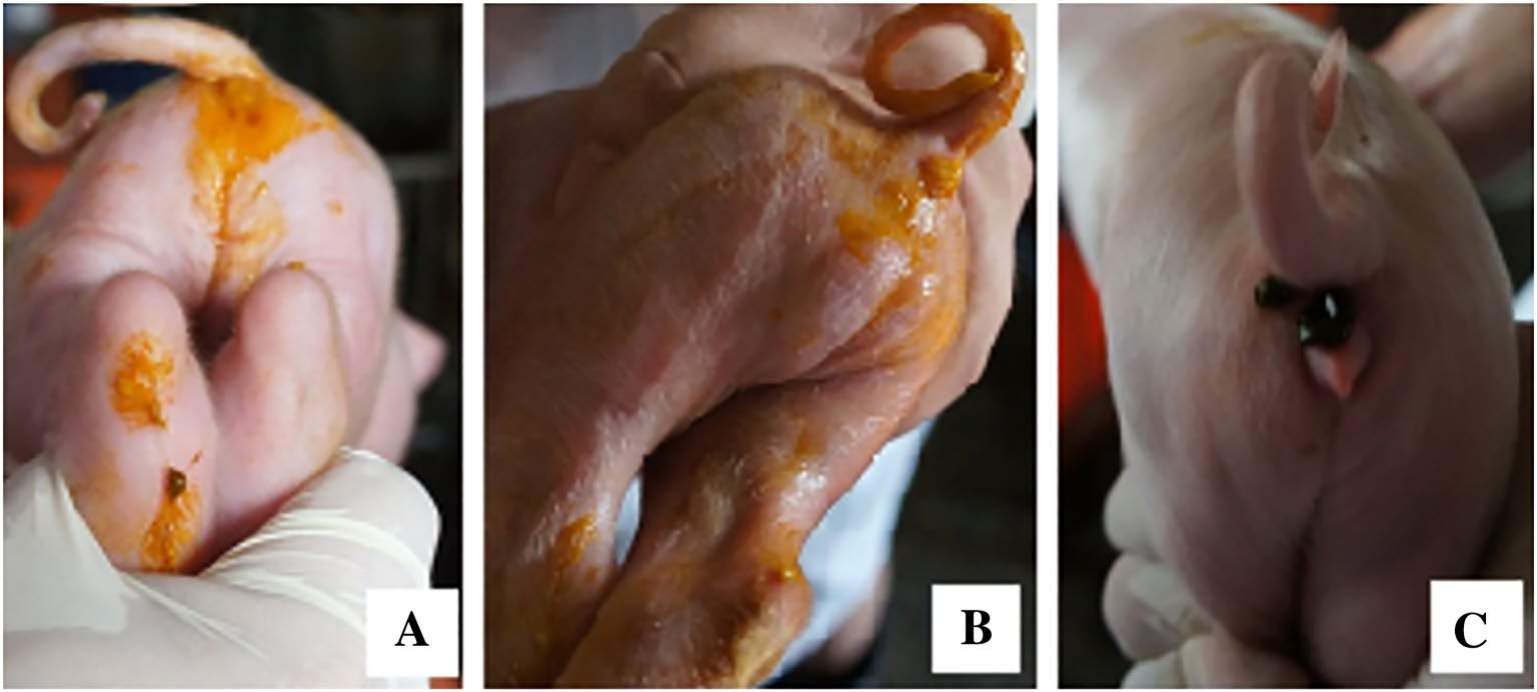
Clinical signs in experimentally infected piglets (**A**, **B**) Hind Quarter of piglets showing signs of watery diarrhea (**C**) healthy animal

**Fig. 4:**
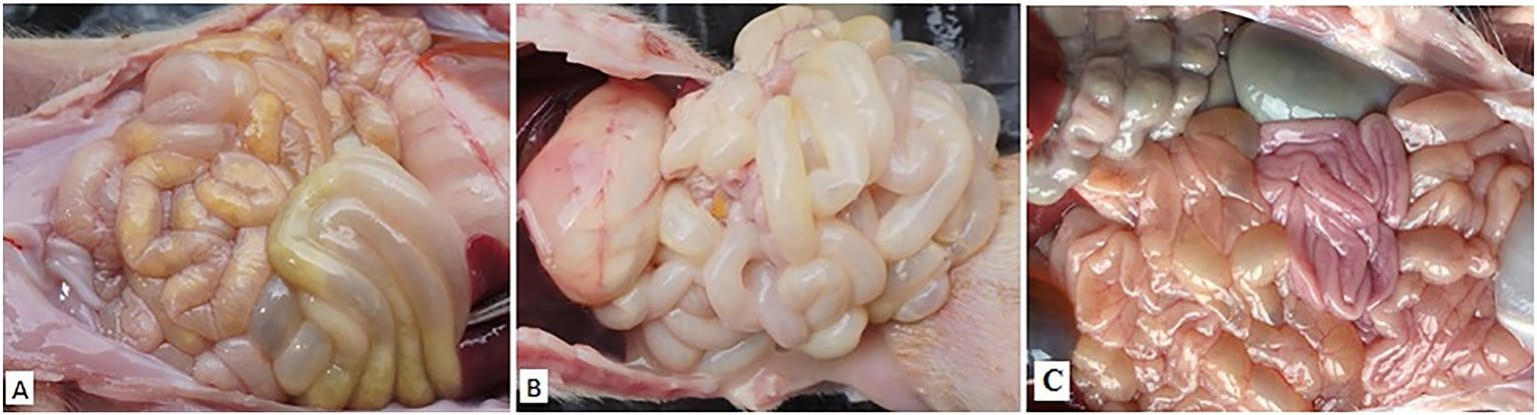
Necropsy examinations of the intestine of piglets. **A**, **B** Inoculated with PEDV-CHYJ130330 strain. The small intestine was thin-walled and contained soft to watery yellowish contents. **C** Inoculated with control medium. No intestinal lesions were found in the uninfected piglets

The CHYJ130330 strain has strong virulence affecting 3 day old piglets after oral administration of 1 ml of 100 TCID_50_ virus solution (morbidity 100%) (Tables 3 and 4). There was vomiting and watery diarrhea in affected piglets that occurs within 36 h (Fig. 3). All 10^4^ TCID_50_ group piglets died of dehydration within 5 days. There was thinning of small intestine with presence of yellowish fluid in affected animals (Fig. 4).

**Table 3:** PEDV shedding in rectal swabs of piglets

Group	TCID_50_/ml	Piglets ID	PEDV RT-PCR result							
			0DPI	1DPI	2DPI	3DPI	4DPI	5DPI	6DPI	7DPI
1	10^4^	1	−	−	+	+	+	×	×	×
		2	−	−	+	+	+	+	×	×
		3	−	−	+	+	+	+	×	×
		4	−	−	+	+	+	+	×	×
		5	−	−	+	+	+	+	+	×
2	10^3^	1	−	−	+	+	+	+	+	+
		2	−	−	+	+	+	+	+	×
		3	−	−	+	+	+	+	+	+
		4	−	−	+	+	+	+	+	+
		5	−	−	+	+	+	+	+	+
3	10^2^	1	−	−	+	+	+	+	+	+
		2	−	−	+	+	+	+	+	+
		3	−	−	+	+	+	+	+	+
		4	−	−	+	+	+	+	+	+
		5	−	−	+	+	+	+	+	−
4	Neg Ctl	1	−	−	−	−	−	−	−	−
		2	−	−	−	−	−	−	−	−
		3	−	−	−	−	−	−	−	−
		4	−	−	−	−	−	−	−	−
		5	−	−	−	−	−	−	−	−

+: RT-PCR positive; −: RT-PCR negative; ×: dead

**Table 4:** Virulence of CHYJ130330 strain 3 day old piglets after oral administration of 1 ml of TCID_50_ virus solutions

Doses	Piglets	Morbidity (%)	Mortality
10^4^TCID_50_	5	100	100%
10^3^TCID_50_	5	100	20%
10^2^TCID_50_	5	100	0

### Histopathological findings

**Fig. 5:**
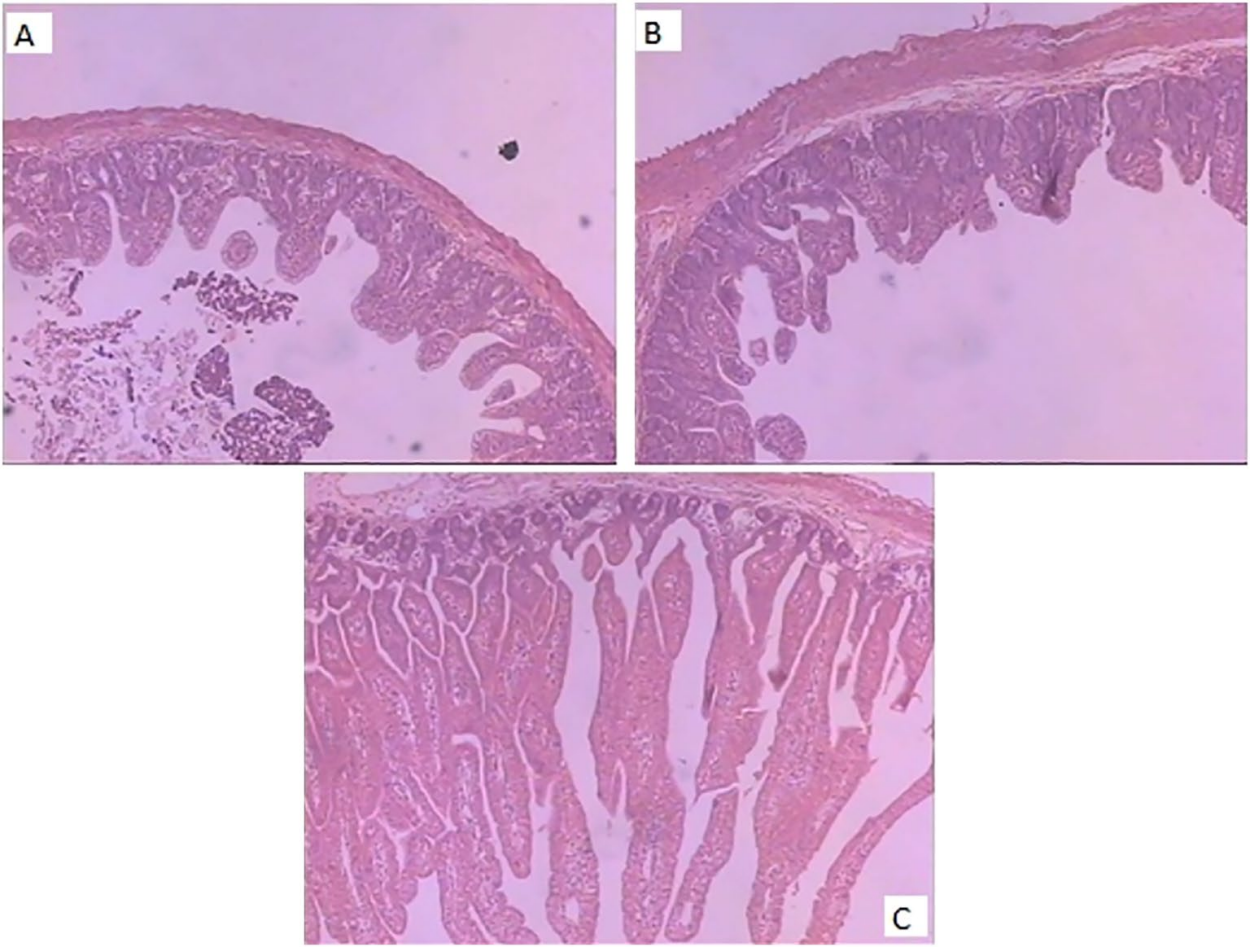
Photomicrographs of intestines of PEDV piglets. **A**, **B** Atrophy of villi with extensive intestinal epithelial degeneration as well as sloughing and necrosis of the villi. **C** No lesions in intestines of uninfected piglets. HE Stain ×100

On histopathology, the intestine showed stunting and fusion of villi (Fig. 5) with necrosis and sloughing of the area (Fig. 5A, B). Mucosal epithelial cells were found necrotic and lysed; having nuclear changes suggesting cell death i.e., pyknosis, karyorrhexis, and karyolysis. The number of intestinal villi was reduced, the length was shortened and the length of the intestine villi and crypt was about 2:1.

### Whole genome sequence comparison and phylogenetic analysis

The CHYJ130330 strain possessed a 28,038 nucleotides long complete genome sequence excluding 3′ poly (A) tail, having unique arrangement as 5′ UTR (1 to 292 nt), a replicase gene (1a/1b) (293 to 20,637 nt), a spike gene (20,634 to 24,794 nt), ORF3 (24,794 to 25,468 nt), an envelope gene (25,449 to 25,679 nt), a membrane gene (25,687 to 26,367 nt), a nucleoprotein gene (26,379 to 27,704 nt), and a 3′ UTR (27,705 to 28,038 nt) [9].

CHYJ130330 strain had 96.5–97.2% nt identity with the pre-2013 strains and 97–99% nt identity with the post-2013 strains reported from China. The exceptions included the CH/ZMDZY/2011(KC196276) strain reported in 2011 having 98.75% identity with the whole genome nt sequence reported from our lab. The whole genome sequences reported from other regions included CV777, vir.DR13/S.Korea, SM98/S.Korea and atten.DR13/S.Korea sharing 96.8%, 97.6%, 96.6 and 97.1% identity with CHYJ130330 respectively while all other selected reference strains shared 98–99% identity with our sequence.

**Fig. 6:**
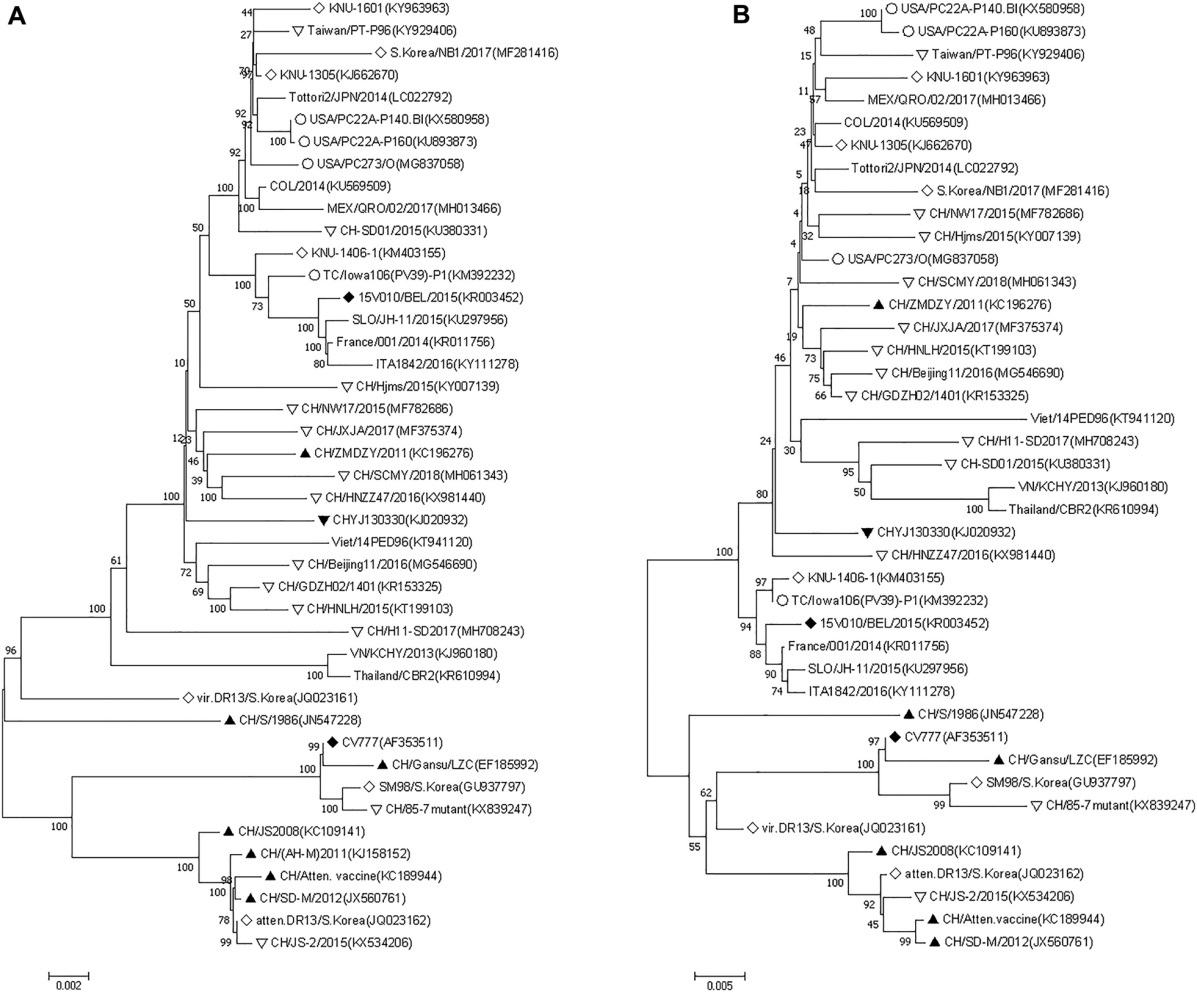

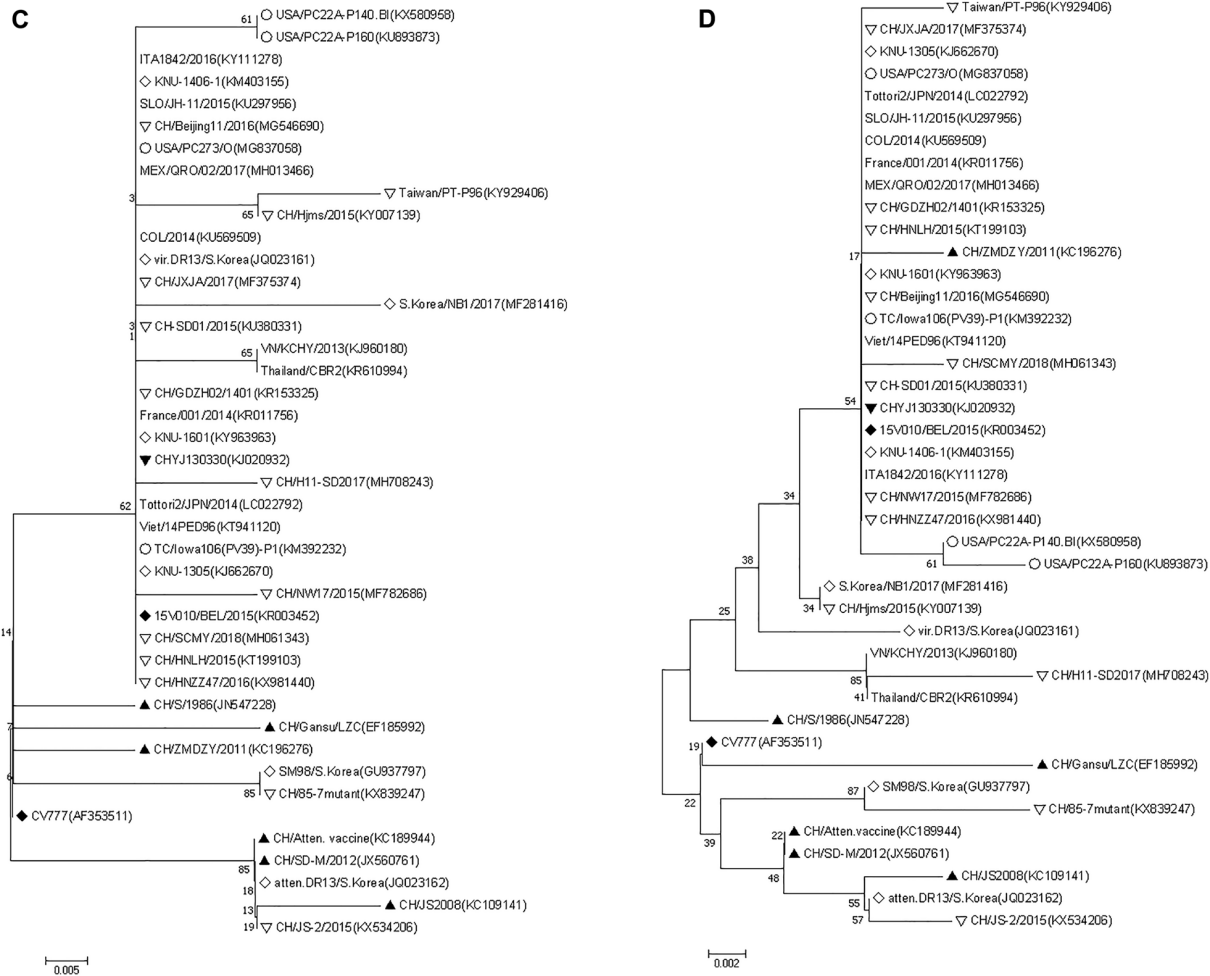

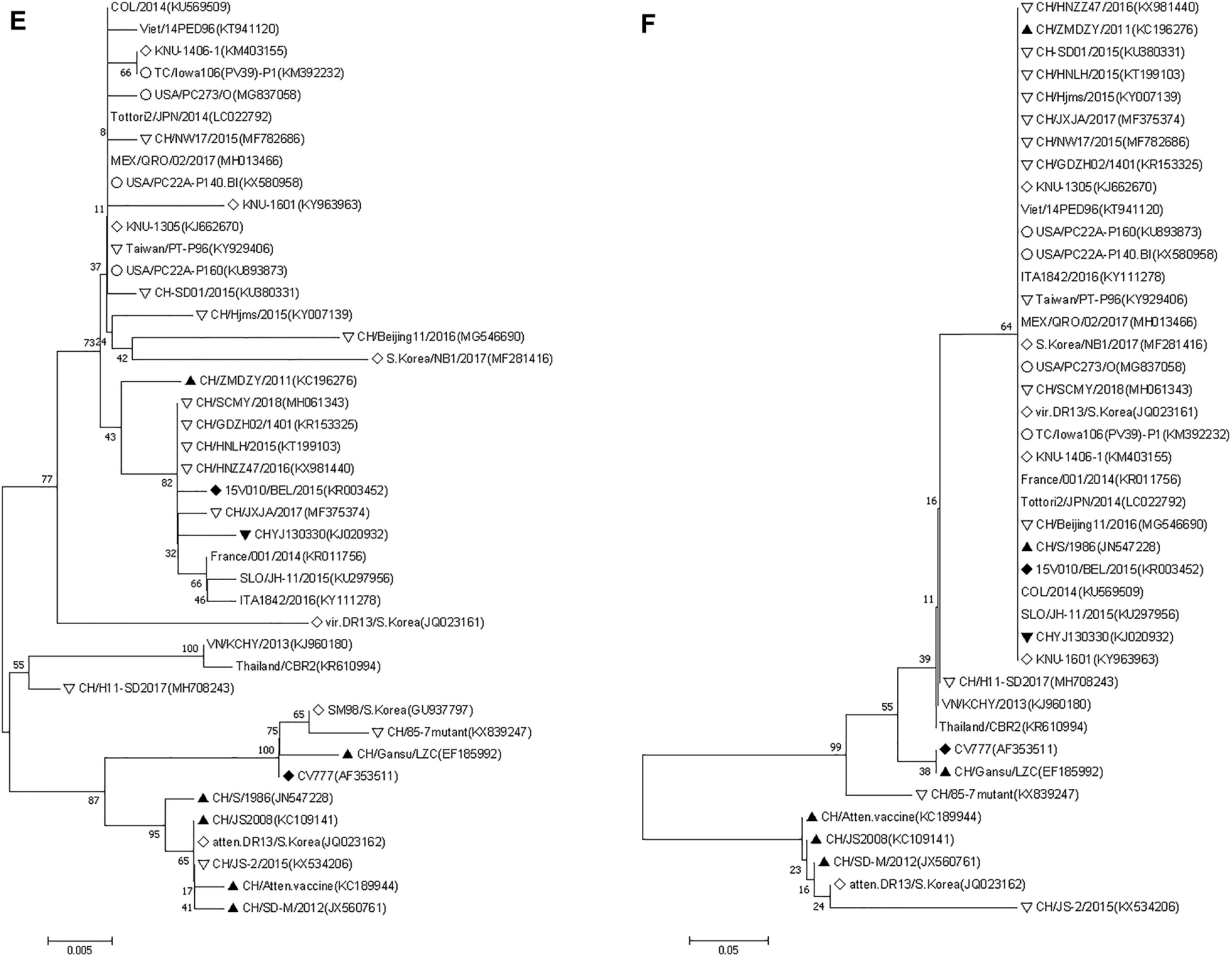
**A**–**F** Phylogenetic trees based on **A** complete genome of 43 PEDVs (**B**–**F**) Amino acid sequence of S glycoprotein gene, envelope, membrane, nucleocapsid and ORF3 genes respectively. The trees were constructed by the distance-based neighbor-joining algorithm using MEGA software. Bootstrap was set in 1000 replicates with a value > 70% to assess the significance of the tree topology. A bar of 0.002/0.005/0.05 indicated nucleotide or amino acid substitutions per site. “white circle” indicated strains from USA, “white diamond” indicated strains from South Korea, “white down-pointing triangle” indicated strains from China after 2013, “black down-pointing triangle” indicated strain from this study, “black diamond” indicated strains from Belgium, “black up-pointing triangle” indicated strains from China before 2013, “No symbol” indicated strains from other parts of world.

The phylogenetic analysis of 43 reference strains including highly virulent CHYJ130330 had clearly distinguished them into two groups namely G1 and G2. G1 had three while G2 was further split into two subgroups respectively. In addition to 1a and 1b subgroup; G1 also had a tentative cluster comprising virulent DR13 (South Korea/1999) and CH/S (China/1986) strains placed in R group (Fig. 6A). The prototype strain of CV777, Korean (attenuated DR13, virulent DR13 and SM98) and earlier Chinese strains (CH/Atten. Vaccine, CH/S, CH/85-7mutant, JS2008, LZC and SD-M/2012) comprised the G1 group. More importantly all the strains reported after 2013 from China till date were fallen in group G2 except CH/JS-2/2015(KX534206).

### Comparative analysis of structural genes

#### Spike (S) protein gene

The full-length of S gene of CHYJ130330 was 4,160 nt in size, which was 8-nt longer than that of the prototype of PEDV CV777 strain. CHYJ130330 strain has 92.7–93.3% aa identity with the pre-2013 strains except CH/ZMDZY/2011(KC196276) reported from central China having 98.1% sequence similarity. The post-2013 PEDV strains have 95.2–98.4% aa identity except CH/85-7mutant (KX839247) and CH/JS-2/2015(KX534206) sharing 92% to 93% similarity reported from China. CHYJ130330 strain shared 93.7% aa identity with CV777, 92.9–94.6% identity with three S. Korean strains which also included the cell adapted PEDV vaccine strain, 95.6–98.7% identity with the other 19 strains of PEDV selected from various regions of the world which also included four strains of PEDV reported from USA.

The S protein targeted phylogenetic tree using 43 strains of PEDV showed two major groups, having two subgroups each. The strain CHYJ130330 belonged to subgroup 2b, which included all other strains reported from China post-2013 except one strain JS-2/2015 which was a part of group (G1). All other strains reported from China pre-2013 belong to group G1; including the attenuated Vaccine strain, KC189944 reported from Hubei, China. The S protein of PEDV strains reported from USA also belongs to Group G2 (Fig. 6B).

### Envelope (E) protein gene

The full-length of E gene of CHYJ130330 was 230 nt, encoding 76-aa protein. CHYJ130330 strain has 94.7–97.4% aa identity with the pre-2013 strains and 98.7–100% aa identity with the post-2013 strains reported from China except 85-7mutant(KX839247), ZL29/2015(KU847996), HNQX-3/14(KR095279) and JS-2/2015(KX534206) strains which have 95.6–97.4% similarity with our sequence. CHYJ130330 has 98.6–100% similarity with all other sequences including CV777. The exceptions include SM98/S.Korea (96%), atten.DR13/S.Korea (95%), NB1/S.Korea (97.4%).

The aa deduced phylogenetic tree depicted two groups, that is, Group 1 and Group 2 (Fig. 6C). The strain CHYJ130330 belonged to group G2, which included all PEDV strains selected in this study reported from China post-2013 along with other globally reported E protein sequences. The G1 group included the CV777, attenuated Vaccine strain (KC189944), 85-7mutant(KX839247) and JS-2/2015(KX534206) and other strains reported from China pre-2013.

### Membrane (M) protein gene

The full-length of M gene of CHYJ130330 was 680 nt, encoding 226 amino acids. M protein of CHYJ130330 strain has 96.9–99.6% aa identity with the pre-2013 and more than 97.3% aa identity with the post-2013 strains reported from China, with many having 100% identity indicating that the sequence of M protein is highly conserved. CHYJ130330 strain shared 98.7% aa identity with CV777, 97.8–100% identity with other reference PEDV strains.

The phylogenetic analysis of M protein confirmed two groups, i.e., Group 1 and Group 2 in the chosen strains (Fig. 6D). Group 2 contained 12 Chinese strains, including CHYJ130330, and US strains. The G1 group includes the CV777, attenuated Vaccine strain (KC189944), JS-2/2015(KX534206) and other strains reported from China pre-2013.

### Nucleocapsid (N) protein gene

The full-length of N gene of CHYJ130330 was 1325 nt. N protein of CHYJ130330 strain has 95.5–96.6% aa identity with the pre-2013 Chinese strains except 98.6% aa identity with ZMDZY(KC196276) strain. The post-2013 strains reported from China have 96.4–99.5% aa identity. In addition to CV777, few S. Korean and Thailand strains and almost all other strains shared 98–99.3% similarity with CHYJ130330. The CV777 has 96.1% while others (S. Korean and Thailand strains) have 95.9–96.8% identity with amino acid sequence of N protein reported in this study (Fig. 6E).

The phylogenetic analysis showed that CHYJ130330 strain is present in the group 1b along with other major nucleoprotein sequences reported from China. CV777, attenuated vaccine strain and predominant sequences reported from China pre-2013 belong to Group (G1).

### ORF3 protein gene

The full-length of ORF3 gene of CHYJ130330 was 674 nt, encoding 224 amino acids. CHYJ130330 strain has 90.1–94.1% aa identity with the pre-2013 strains except CH/S/1986(JN547228) and ZMDZY/2011(KC196276) which have 99.5% and 99.1% homology respectively with our strain. The post-2013 strains have 96.4–100% aa identity with CHYJ130330 except CH/JS-2/2015(KX534206) from Shanghai sharing 86.8% identity with ORF3 protein.

CHYJ130330 strain shared 96% aa identity with CV777, more than 98% identity with other reference strains from various parts of the world except VN/KCHY/2013(KJ960180), atten.DR13/S.Korea(JQ023162) and Thailand/CBR2(KR610994) sharing 96%, 91.2% and 96.4% homology respectively (Fig. 6F).

## Discussion

Until 2010, Porcine epidemic diarrhea was considered as a sporadic enteric viral disease in China causing enteritis and diarrhea in piglets seasonally [13]. However, towards the end of 2010, the disease appeared with more severity, morbidity and mortality. Large scale outbreaks were reported at pig farms in Southern China with piglets having acute diarrhea and severe dehydration. The disease was highly contagious and soon spread all over the country posing major economic threat to the swine industry [14, 15]. The outbreaks continued in the following years at various commercial pig farms however, it was established by that time, that GII variant strains varying genetically from classical ones were the main reason behind repeated outbreaks and vaccination failure [1]. The PEDV vaccines including classical PEDV strain CV777, the inactivated bivalent transmissible gastroenteritis virus (TGEV) and PEDV vaccine (1999 to present) and the attenuated bivalent TGEV and PEDV vaccine (2003–2006) have been widely used, the PED outbreaks still occurred in vaccinated herds with high mortality in suckling piglets, highlighting the inadequate immune protection using classical vaccine strains against variant GII genogroup [16]. This mutation in the PEDV genome, which gave rise to the variant strains, was a major challenge for the prevention and control of this disease worldwide. These antigenic variations that gave rise to highly virulent variant strains were also reported in various serological cross reactivity assays [17, 18]. It was therefore necessary to study biological characteristics, pathogenicity index and evolutionary analysis of the field prevalent strains.

In this study, a wild-type PEDV strain CHYJ130330 (GenBank accession no. KJ020932), isolated from diarrheal piglet belonging to a GII genogroup was isolated and propagated in a Vero cell line, which demonstrated obvious cytopathic effects characterized by cell fusion and syncytium formation after 24 h of incubation. It was found that CHYJ130330 strain has strong virulence, affecting 3 day old piglets, when orally administered with 1 ml of 100 TCID50 virus solutions (morbidity 100%). Vomiting and watery diarrhea was observed within 36 h in affected piglets. There was thinning of the inner wall of intestine along with presence of yellowish fluid and all 10^4^ TCID50 group piglets died of dehydration within 5 days. Experimental infections with highly virulent PEDV strains in different studies resulted in consistent outcomes [19–21]. The histopathological examination revealed changes in the mucosal epithelial cells along with reduction, shortening and fusion of the intestinal villi [22]. An effort has constantly being carried out with some initial experimentation having good results to develop a live attenuated vaccine from CHYJ130330 strain, which gives more promising results against GII genogroup strains circulating in China.

PEDV strains on the basis of phylogeny have two groups, GI (Classical) and GII (Variant). Each group has further two subgroups; GI-a, GI-b, GII-a, GII-b (Fig. 6A). The overall results for phylogeny and percent identity resembles those of many studies with minor exceptions indicating a similar pattern of disease occurrence with both classical and mutant strains prevailing across many countries in the world [23]. The Classical Chinese strains (LZC, CH/S etc.), as well as the prototype strains (virulent CV777 and DR13) all have GI-a subgroup, while predominant strains in GI-b subgroup are cell-culture adapted vaccine strains (attenuated CV777 and DR13) and other pandemic classical strains (AH-M etc.). The group GI involved in sporadic outbreaks, therefore included mainly classical strains i.e., JS2008(KC109141), CH/S(JN547228), LZC(EF185992), SD-M(JX560761), from China; virulent DR13(JQ023161), SM98(GU937797) from South Korea. It was later in 2010, when pandemic strains were reported in some other countries including South Korea and Thailand; however the infection rate in these outbreaks as reported was slow. The disease severity and infection rate was however higher in China affecting pigs of all ages during that time period [15]. Furthermore, the strains reported to be involved were not only pandemic variant strains (GII genogroup) [1, 24] but also included the pandemic classical strains. Our strain which was reported in 2013 along with many other strains currently prevalent in China also belongs to Group II [9].

The percentage similarity based on whole genome indicated that our strain CHYJ130330 (KJ020932) showed the highest nucleotide identity (> 98.5) with about 50% strains reported all over the China during this decade. The strain reported by our group belonged to the Southern China (Guangdong province). Most of the strains having higher similarity were reported after 2013, and they are all variant strains belonging to genogroup II [25]. Among the sequences selected in this study from China, there was one sequence which was collected in 2011 from Central China, Henan province; this sequence has 98.75% similarity with our sequence. It may be concluded from this that the variant strains were circulating all over China after 2010 and may be the Guangdong province having highest population of pigs was not responsible for the origin of variant PEDV strains. PEDV was reported for the first time in USA in 2013. The percentage similarity of our sequence, sequences from USA, Europe and other Asian countries indicated the high prevalence of Variant strains (GII group) worldwide. The field PEDV strains SD-M and JS2008 including atten. vaccine DR13 strain and Chinese vaccine strain (KC189944) were found clustered independently in a tree having a 24-nt deletion in nsp 3 (ORF1a) and 49-nt deletion in ORF3 (C terminus). This uniqueness suggested their origin from same source and also indicates recombination which might have occurred between the vaccine strains and above mentioned field PEDV strains.

It is known that S protein plays an important role in entry of virus and induction of neutralizing antibodies in natural hosts, therefore it was a main target for development of an effective vaccine [26, 27]. The percentage identity showed higher similarity among all sequences reported after 2010 including our sequence, as compared to prototype and classical strains including CV777(AF353511), CH/S(JN547228), CH/Atten.vaccine(KC189944), JS2008(KC109141), LZC(EF185992), SD-M(JX560761), vir.DR13(JQ023161), SM98(GU937797), atten.DR13(JQ023162), 85-7mutant(KX839247), JS-2/2015(KX534206). The amino acid comparison among our sequence (CHYJ130330) (As representative of GII group), CV777 and PEDV attenuated vaccine revealed significant genetic variations as reported earlier [6]. A high nucleotide and amino acid variation was observed among these reference strains especially in the S region [28]. These genetic variations therefore made variant GII group strains different from the earlier classical PEDV strains including CV777 and attenuated strains. This might also explained the variable trend of large scale outbreaks during this decade as compared to sporadic tendency of disease found before 2010. It also explained possible reason of inadequate protection in pigs vaccinated with classical field strain based vaccines including CV777 on swine farms.

The E and M protein sequences were more conserved and identical, showing CHYJ130330 more similar to the variant group. The phylogenetic analysis predicted the vir.DR13/S.Korea, USA strains and all variant Chinese strains (GII genogroup) reported after 2010 to be having the same evolutionary origin. Compared to CV777, CHYJ130330 shared 1 and 3-aa substitutions respectively i.e., for E protein at residue 65(R65Q) and for M protein at 13(E13Q), 42(V42A) and 213(A213S).

It is known that changes in the ORF3 are due to the virus adaptation during serial passage in the cells [29]. Accordingly in this study, the deduced amino acid sequence of CV777, earlier strains; SM98 and LZC, and other cell adapted strains SD-M etc. including atten. DR13 were all in group I-b while other Chinese strains (CHYJ130330) including CH/S, Korean strain (Vir. DR13) and US strains comprised groups (GI-a, GII-a, GII-b) [30]. Compared to CV777, CHYJ130330 shared 9-aa substitutions, at residues 21 (V21A), 54 (V54I), 79 (V79I), 80 (F80V), 85 (L85I), 92 (L92F), 101 (A101T, from hydrophobic polarity A to hydrophilic polarity T), 166 (N166S, from hydrophobic N to hydrophilic S) and 182(H182Q) in the ORF3 protein. These unique substitutions were reported earlier in some other studies also [5]. When the Aa sequences of PEDV N protein were subjected to multiple sequence alignment, it divides them into two groups; each group having two subgroups. CHYJ130330 strain when compared to CV777 and Atten. Vaccine strain shared 17- and 21-aa substitutions respectively.

To summarize, a highly virulent novel strain was isolated and identified which gives us insight into the molecular characteristics, pathogenicity assessment genetic/phylogenetic variations of the Guangdong (Province having highest pig population) field strain associated with outbreaks. The comparison of full length genome and the structural proteins revealed variations signifying that PEDV variant strains are still the main source of outbreaks in spite of continuous vaccination. It is evident from this study that Chinese strains displayed significant level of mixing with the strains reported from other countries also. The strain identified in this study was also adapted successfully to Vero cell line and has high virulence in piglets. PEDV CHYJ130330 has strong virulence and is a more popular clinical strain in recent years, which has the potential to be developed into PEDV vaccine.

## Data Availability

Not applicable.
